# Venous thromboembolism in metastatic urothelial carcinoma or variant histologies: incidence, associative factors, and effect on survival

**DOI:** 10.1002/cam4.986

**Published:** 2016-12-20

**Authors:** Jorge D. Ramos, Martin F. Casey, Simon J. Crabb, Aristotelis Bamias, Lauren C. Harshman, Yu‐Ning Wong, Joaquim Bellmunt, Ugo De Giorgi, Sylvain Ladoire, Thomas Powles, Sumanta K. Pal, Guenter Niegisch, Federica Recine, Ajjai Alva, Neeraj Agarwal, Andrea Necchi, Ulka N. Vaishampayan, Jonathan E. Rosenberg, Matthew D. Galsky, Evan Y. Yu, RISC Investigators

**Affiliations:** ^1^ University of WashingtonSeattleWashington; ^2^ Icahn School of Medicine at Mount SinaiNew YorkNew York; ^3^ University of SouthamptonSouthamptonEngland; ^4^ National and Kapodistrian University of AthensAthensGreece; ^5^ Dana Farber Cancer InstituteBostonMassachusetts; ^6^ Fox Chase Cancer CenterPhiladelphiaPennsylvania; ^7^ Hospital del MarBarcelonaSpain; ^8^ Istituto Scientifico Romagnolo per lo Studio e la Cura dei Tumori (IRST) IRCCSMeldolaItaly; ^9^ Georges François Leclerc CenterDijonFrance; ^10^ Université de BourgogneDijonFrance; ^11^ Barts and the London School of MedicineLondonEngland; ^12^ City of HopeDuarteCalifornia; ^13^ Heinrich‐Heine‐UniversityDüsseldorfGermany; ^14^ University of MichiganAnn ArborMichigan; ^15^ University of UtahSalt Lake CityUtah; ^16^ Fondazione IRCCS Istituto Nazionale dei TumoriMilanItaly; ^17^ Karmanos Cancer InstituteDetroitMichigan; ^18^ Memorial Sloan Kettering Cancer CenterNew YorkNew York

**Keywords:** Urothelial, bladder cancer, chemotherapy survival, venous thromboembolism

## Abstract

Venous thromboembolism (VTE) is common in cancer patients. However, little is known about VTE risk in metastatic urothelial carcinoma or variant histologies (UC/VH). We sought to characterize the incidence, associative factors, including whether various chemotherapy regimens portend different risk, and impact of VTE on survival in metastatic UC/VH patients. Patients diagnosed with metastatic UC/VH from 2000 to 2013 were included in this multicenter retrospective, international study from 29 academic institutions. Cumulative and 6‐month VTE incidence rates were determined. The association of first‐line chemotherapy (divided into six groups) and other baseline characteristics on VTE were analyzed. Each chemotherapy treatment group and statistically significant baseline clinical characteristics were assessed in a multivariate, competing‐risk regression model. VTE patients were matched to non‐VTE patients to determine the impact of VTE on overall survival. In all, 1762 patients were eligible for analysis. There were 144 (8.2%) and 90 (5.1%) events cumulative and within the first 6 months, respectively. VTE rates based on chemotherapy group demonstrated no statistical difference when gemcitabine/cisplatin was used as the comparator. Non‐urotheilal histology (SHR: 2.67; 95% CI: 1.72–4.16, *P *< 0.001), moderate to severe renal dysfunction (SHR: 2.12; 95% CI: 1.26–3.59, *P *= 0.005), and cardiovascular disease (CVD) or CVD risk factors (SHR: 2.27; 95% CI: 1.49–3.45, *P *= 0.001) were associated with increased VTE rates. Overall survival was worse in patients with VTE (median 6.0 m vs. 10.2 m, *P* < 0.001). Thus, in metastatic UC/VH patients, VTE is common and has a negative impact on survival. We identified multiple associated potential risk factors, although different chemotherapy regimens did not alter risk.

## Introduction

Cancer patients are at increased risk of developing a venous thromboembolism (VTE). It is now estimated that approximately 20–30% of first VTE in patients are cancer associated 1. Improved survival outcomes across multiple malignancies, increasing use of central venous catheters, and better imaging modalities to detect venous thrombosis have led to a rising incidence of cancer‐associated thrombosis over time 2. Importantly, VTEs have been shown to have a negative impact on survival 3. In addition, cancer patients with a VTE have increased morbidity from anticoagulation, with a higher risk of recurrent VTE and major bleeding compared to noncancer patients with VTE 4.

Patient‐related characteristics such as a high body mass index (BMI), elevated white blood cell (WBC) and platelet count, and low hemoglobin have been incorporated into the Khorana score, a validated, predictive model of VTE risk in patients who are initiating chemotherapy 5. When looking at specific cancer primary sites, the most aggressive malignancies, such as pancreatic cancer, have tended to have the greatest VTE risk 6. Finally, there is a mounting body of evidence for chemotherapy as an independent risk factor for VTE, with the strongest data for cisplatin 7, 8, 9. Moreover, some studies have indicated especially high thromboembolic rates (arterial and venous) in patients treated with the combination of gemcitabine and cisplatin (GC) 7, 10, 11, 12.

Despite the growing amount of data on cancer‐associated thrombosis, there is limited literature assessing the incidence and risk factors for VTEs in urothelial carcinoma or variant histologies (UC/VH), especially in the metastatic setting. Additionally, UC/VH have been underrepresented in previous VTE studies. In the patient cohort utilized to develop the Khorana score, genitourinary malignancies (excluding prostate cancer) were grouped with other primary tumor sites which only represented approximately 10% of the study population, yet they are still considered a high‐risk primary tumor site and given 1 point in the model 5. Likewise, in the largest randomized, prospective clinical trial assessing primary thromboprophylaxis across multiple solid tumors, bladder cancer only accounted for 2% of the patient population 13. Given the dearth of information on VTE in patients with metastatic UC/VH, we conducted a retrospective cohort study to better understand the incidence and risk factors associated with VTE, whether chemotherapy (in particular the combination of GC) increases VTE risk, and the impact of VTE on survival.

## Methods

### Study design and patient population

The Retrospective International Study of Cancers of the Urothelium (RISC) is a multicenter study of the management and outcomes of patients with cancers of the urothelial tract with at least muscle‐invasive disease (clinical T‐classification of ≥2). Baseline characteristics, laboratory, pathology, treatment, and outcome data were collected and compiled via a secure, password‐protected electronic data capture platform from 29 international centers as previously described 14. The study was approved by the institutional review board at all participating institutions.

Patients diagnosed with metastatic disease of the bladder, renal pelvis, ureter, and urethra from 1 January 2000 through 31 December 2013 were eligible for analysis. Urothelial, adenocarcinoma, micropapillary, sarcomatoid, small cell, and squamous histologies were included for analysis. In patients with mixed histology tumors, the predominant histologic pattern of the tumor was utilized for categorization. Patients without a known date of diagnosis, date of last follow‐up, or VTE data were excluded from the study. First‐line chemotherapy regimens were subdivided into six groups for analysis: (1) GC, (2) gemcitabine and carboplatin, (3) cisplatin combination (excluding GC), (4) nonplatinum regimens, (5) carboplatin or oxaliplatin (excluding gemcitabine and carboplatin), and (6) no chemotherapy. Specific chemotherapy regimens within the chemotherapy subgroups are listed in supplementary Table 1. Based on previous data suggesting particularly high rates of thromboembolic events with GC, we hypothesized that there would be more VTEs in this treatment group when compared to other treatment groups. Additionally, GC is a commonly used regimen for advanced disease, therefore, GC was chosen as the reference for comparison to the other chemotherapy treatment groups 15. Other baseline patient, tumor, and treatment‐related characteristics were assessed for their impact on VTE incidence (see full list in Table S2).

**Table 1:** Patient characteristics

	*N* (%)
Total	1762 (100)
Age	
<40	15 (0.9)
40–64	683 (38.8)
>65	1039 (59.0)
Unknown	25 (1.4)
Gender	
Male	1365 (77.5)
Female	390 (22.1)
Unknown	7 (0.4)
Race	
White	1595 (90.5)
Other	157 (8.9)
Unknown	10 (0.6)
Primary tumor location	
Bladder	1462 (83.0)
Other (renal pelvis, ureter, or urethra)	265 (15.0)
Unknown	35 (2.0)
Histology	
Urothelial	1525 (86.5)
Non‐urothelial	183 (10.4)
Unknown	54 (3.1)
Primary treatment	
Surgery^a^	593 (33.7)
Surgery^a^ with perioperative chemotherapy	422 (24.0)
Radiation	117 (6.6)
Radiation with concurrent chemotherapy	89 (5.1)
None/Unknown	541 (30.7)

a Encompasses patients who underwent a radical cystectomy, nephroureterectomy, nephrectomy, ureterectomy, or urethrectomy as their primary treatment modality.

### Statistical analysis

Cumulative and 6‐month absolute VTE incidence rates were calculated from the date of diagnosis of metastatic disease to the VTE date. Each baseline and treatment‐related factor was assessed, using competing‐risk regressions, for association with the development of VTE. Multiple imputations with chained equations were performed to address any missing data. First‐line chemotherapy group and any statistically significant covariates in the univariate analysis (*P* ≤ 0.05) were evaluated in a multivariate, competing‐risk regression model. Subdistribution hazard ratios (SHR) were calculated for each factor.

To assess survival, a nested case–control analysis was performed where VTE patients (cases) were matched to non‐VTE patients (controls). Cases and controls were matched based on time from date of diagnosis of metastatic disease to date of VTE diagnosis of the case, age, gender, race, and whether the patient received chemotherapy. Patients were also matched based on ECOG performance status and presence of liver metastases as these factors have been previously demonstrated to be poor prognostic features in patients with bladder cancer 16. All attempts were made to match five controls to one case, however, because of the number of variables utilized, 5:1 matching could not be achieved for all strata. In those cases, the highest degree of matching possible was employed. Overall survival was calculated from the time of the VTE event. The log‐rank test was utilized to compare survival between cases and controls. All statistical analyses were performed using Stata 13/IC 13.1.

## Results

### Study population and patient characteristics

**Figure 1:**
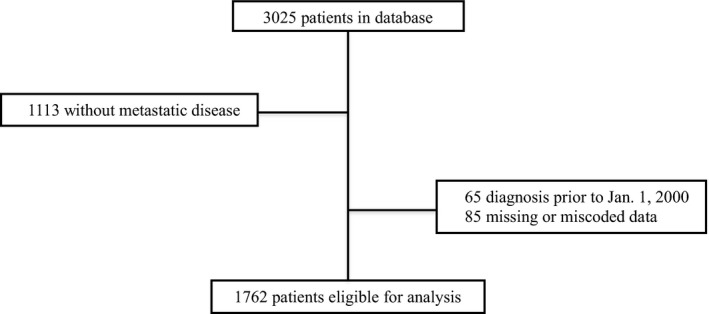
Flowchart of patient inclusion and exclusion criteria.

The RISC database is comprised of 3025 patients. In all, 1912 patients were diagnosed with metastatic disease (Fig. 1). Of these, 65 patients were diagnosed prior to 1 January 2000 and thus excluded from the analysis. An additional 85 patients were excluded due to missing or miscoded date of diagnosis, last follow‐up, or VTE data to arrive at a final cohort of 1762 patients eligible for analysis. Baseline characteristics of the entire study cohort are outlined in Table 1. The majority of the patients were >65 years of age (59.0%), male (77.5%), and white (90.5%). The predominant histologic subtype was urothelial, representing 86.5% of cases. Most patients (63.3%) had localized disease (T2–T4, N0, M0) at the time of cancer diagnosis. Node‐positive (any T, N1–N3, M0) and metastatic disease (any T, any N, M1) accounted for 14.6% and 22.1%, respectively. The median number of cycles of first‐line chemotherapy was four.

### Incidence of venous thromboembolism and association with clinical characteristics

Unadjusted VTE incidence rates based on patient demographics and clinical characteristics of the 1762 patients are shown in Table 2. Altogether, there were 144 VTEs with 90 of them occurring in the first 6 months. The cumulative and 6‐month VTE incidence rate was 8.2% (95% CI: 6.9–9.6%) and 5.1% (95% CI: 4.1–6.2%), respectively. The cumulative VTE rate, when calculated as a function of time, was 7.5 events per 100 person‐years. In the univariate analysis, cumulative VTE incidence was statistically significantly increased in patients with cardiovascular (CVD) or CVD risk factors (*P* < 0.001), moderate to severe renal dysfunction (*P *= 0.001), non‐urothelial tumor histology (*P* < 0.001), and those whose primary tumor was treated with radiation therapy (*P *= 0.02). No other clinical characteristics were statistically significant.

**Table 2:** Unadjusted incidence rates of venous thromboembolic events by clinical characteristics.^g^

	VTE/*N*	Incidence rate (95% CI)^a^	Absolute rate (95% CI)	*P*‐value^b^
Total	144/1762	7.5 (6.4–8.8)	8.2 (6.9–9.6)	–
Age				
<40	1/15	3.6 (0.5–25.7)	6.7 (0.2–31.9)	0.10^c^
40–64	65/683	8.0 (6.3–10.2)	9.5 (7.4–12.0)	
>65	77/1039	7.3 (5.8–9.1)	7.4 (5.9–9.2)	
Unknown	1/25	4.2 (0.6–30.1)	4.0 (0.1–20.4)	
Gender				
Male	108/1365	7.2 (6.0–8.7)	7.9 (6.5–9.5)	0.23
Female	36/390	8.8 (6.4–12.2)	9.2 (6.5–12.6)	Ref
Unknown	0/7	0.0 (…)	0.0 (0.0‐41.0)^d^	–
Race				
White	127/1595	7.2 (6.1–8.6)	8.0 (6.7–9.4)	Ref
Other	15/142	9.7 (5.8–16.1)	9.6 (5.4–15.3)	0.20
Unknown	2/10	18.1 (4.5–72.4)	20 (2.5–55.6)	–
Leukocyte count (/*μ*L)				
≤11,000	93/949	7.7 (6.3–9.4)	9.8 (8.0–11.9)	Ref
>11,000	21/222	9.9 (6.5–15.2)	9.5 (6.0–14.1)	0.61
Unknown	30/591	6.1 (4.2–8.7)	5.1 (3.5–7.2)	–
Platelet count (/*μ*L)				
<350,000	78/804	7.6 (6.1–9.4)	9.7 (7.7–12.0)	Ref
≥350,000	36/352	9.8 (7.0–13.5)	10.2 (7.3–13.9)	0.62
Unknown	30/606	5.8 (4.0–8.3)	5.0 (3.4–7.0)	–
Hemoglobin (g/dL)				
<10	20/171	13.5 (8.7–21.0)	11.7 (7.3–17.5)	0.37
≥10	95/984	7.6 (6.3–9.3)	9.7 (7.9–11.7)	Ref
Unknown	29/607	5.5 (3.8–7.9)	4.8 (3.2–6.8)	–
Body mass index				
<35	99/907	9.4 (7.7–11.4)	10.9 (9.0–13.1)	Ref
≥35	6/56	6.1 (2.8–13.7)	10.7 (4.0–21.9)	0.99
Unknown	39/799	5.1 (3.7–7.0)	4.9 (3.5–6.6)	–
CVD or CVD risk factors^e^				
No	48/794	4.8 (3.6–6.4)	6.0 (4.5–7.9)	Ref
Yes	96/968	10.4 (8.5–12.7)	9.9 (8.1–12.0)	<0.001
Moderate to severe renal dysfunction^f^				
No	122/1605	6.9 (5.8–8.2)	7.6 (6.4–9.0)	Ref
Yes	22/157	14.6 (9.6–22.2)	14.0 (9.0–20.4)	0.001
ECOG performance status				
0	29/345	5.2 (3.6–7.6)	8.4 (5.7–11.8)	Ref
1	63/526	9.9 (7.8–12.7)	12 (9.3–15.1)	0.22
2+	16/193	11.9 (7.3–19.5)	8.3 (4.8–13.1)	0.78
Unknown	36/698	6.0 (4.3–8.3)	5.2 (3.6–7.1)	–
Primary tumor location				
Bladder	121/1462	7.9 (6.6–9.4)	8.3 (6.9–9.8)	Ref
Other (renal pelvis, ureter, or urethra)	21/265	6.1 (4.0–9.3)	7.9 (5.0–11.9)	0.58
Unknown	2/35	6.2 (1.5–24.7)	5.7 (0.7–19.2)	–
Histology				
Urothelial	105/1525	6.2 (5.1–7.5)	6.9 (5.7–8.3)	Ref
Non‐urothelial	34/183	18.9 (13.5–26.5)	18.6 (13.2–25.0)	<0.001
Unknown	5/54	12.3 (5.1–29.7)	9.3 (3.1–20.3)	–
Liver metastases				
No	119/1421	7.3 (6.1–8.7)	8.4 (7.0–9.9)	Ref
Yes	24/324	9.1 (6.1–13.6)	7.4 (4.8–10.8)	0.66
Unknown	1/17	4.4 (0.6–31.5)	5.9 (0.1–28.7)	–
Primary tumor radiation therapy				
No	113/1450	6.9 (5.8–8.3)	7.8 (6.5–9.3)	Ref
Yes	23/206	14.1 (9.3–21.2)	11.2 (7.2–16.3)	0.02
Unknown	8/106	6.7 (3.3–13.3)	7.5 (3.3–14.3)	–
First‐line chemotherapy				
Gemcitabine and cisplatin (GC)	50/456	7.7 (5.8–10.2)	11.0 (8.2–14.2)	Ref
Gemcitabine and carboplatin	34/346	9.2 (6.6–12.9)	9.8 (6.9–13.5)	0.81
Cisplatin combination (excluding GC)^f^	14/202	4.7 (2.8–7.9)	6.9 (3.8–11.4)	0.12
Nonplatinum based	22/258	8.1 (5.4–12.4)	8.5 (5.4–12.6)	0.67
Carboplatin or oxaliplatin	8/75	9.4 (4.7–18.7)	10.7 (4.7–19.9)	0.99
No chemotherapy	13/403	5.6 (3.2–9.6)	3.2 (1.7–5.5)	0.008
Unknown	3/22	22.6 (7.3–70.2)	13.6 (2.9–34.9)	–

a VTEs/100 person‐years.

b *P*‐values obtained with univariate competing‐risk regressions using the imputed dataset. Univariate regressions were run to assess for significance and determine inclusion in the final multivariate model. The *P*‐values calculated are based on absolute VTE incidence rates.

c Univariate analysis on age performed with age as a continuous variable.

d One‐sided, 97.5% confidence interval.

e CVD, cardiovascular disease; CVD encompasses coronary artery disease, peripheral vascular disease, a history of myocardial infarction, or a cerebrovascular accident; CVD risk factors analyzed include diabetes mellitus, hypertension, and hyperlipidemia.

Investigator‐designated moderate to severe renal dysfunction.

f 76% received methotrexate, vinblastine, doxorubicin, cisplatin (MVAC).

g Several additional variables were analyzed and not found to be significant in the univariate analysis, including albumin, presence of lymph node metastases, history of VTE, surgery within 2 months of diagnosis of metastatic disease and perioperative chemotherapy, and number of cycles of first‐line chemotherapy. They are omitted from this table for brevity

### Incidence of venous thromboembolism and first‐line chemotherapy

**Figure 2:**
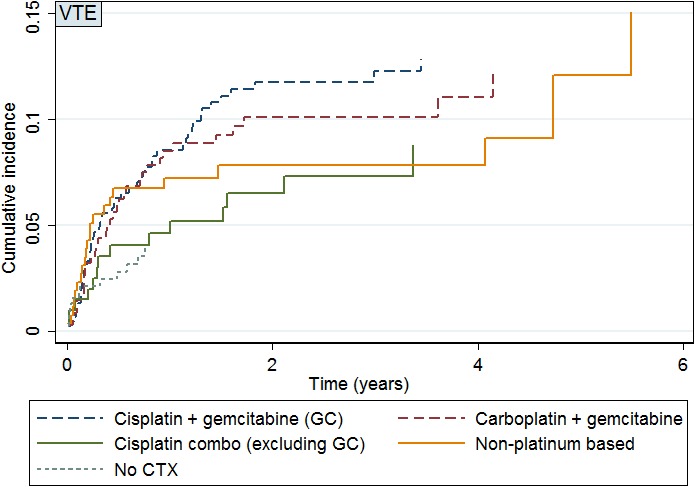
Absolute cumulative incidence of venous thromboembolic events based on first‐line chemotherapy regimen. CTX, chemotherapy; VTE, venous thromboembolism. *Carboplatin and oxaliplatin regimens (excluding gemcitabine and carboplatin) not shown due to comparatively smaller sample size, representing 4.3% of the cohort.

The cumulative incidence of VTE in patients who received chemotherapy was 9.6% (95% CI: 8.1–11.3%). Unadjusted absolute, cumulative VTE incidence rates based on first‐line chemotherapy regimen are shown in Table 2. The absolute cumulative incidence over time for each chemotherapy regimen is shown in Figure 2. The highest absolute cumulative VTE incidence was seen in patients who received GC at 11.0% (95% CI: 8.2–14.2%). Gemcitabine and carboplatin and cisplatin‐containing regimens accounted for an absolute cumulative incidence of VTE of 9.8% (95% CI: 6.9–13.5%) and 6.9% (95% CI: 3.8–11.4%), respectively. Patients who did not receive chemotherapy had an absolute VTE incidence rate of 3.2% (95% CI: 1.7–5.5%).

### Multivariate analysis of venous thromboembolism risk

In addition to chemotherapy treatment group and statistically significant variables in the univariate analysis, age, gender, and race were included as demographic variables in the multivariate model. CVD or CVD risk factors (SHR: 2.27; 95% CI 1.49–3.45, *P *= 0.001), moderate to severe renal dysfunction (SHR: 2.12; 95% CI 1.26–3.59, *P *= 0.005), and non‐urothelial histology (SHR: 2.67; 95% CI: 1.72–4.16, *P* < 0.001) were associated with an increased risk of VTE (Table 3). Patients who were treated with radiation therapy to the primary tumor did not have a statistically significantly greater VTE risk in the multivariate model (SHR: 1.42; 95% CI: 0.83–2.41, *P *= 0.20). Using GC as the reference, there was no statistically significant difference in VTE incidence rates based on chemotherapy regimen. However, patients who did not receive systemic chemotherapy had a decreased incidence of VTE compared to those treated with GC (SHR: 0.32; 95% CI: 0.16–0.61, *P *= 0.001).

**Table 3:** Multivariate, competing‐risk regression analysis of thromboembolic risk based on clinical characteristics

	SHR	95% CI	*P*‐value
CVD or CVD risk factors^a^			
No	1.0	Ref	Ref
Yes	2.27	1.49–3.45	0.001
Moderate to severe renal dysfunction^b^			
No	1.0	Ref	Ref
Yes	2.12	1.26–3.59	0.005
Histology			
Urothelial	1.0	Ref	Ref
Non‐urothelial	2.67	1.72–4.16	<0.001
Primary tumor radiation therapy			
No	1.0	Ref	Ref
Yes	1.42	0.83–2.41	0.20
First–line chemotherapy^c^			
Gemcitabine and cisplatin (GC)	1.0	Ref	Ref
Gemcitabine and carboplatin	0.86	0.53–1.39	0.53
Cisplatin combination (excluding GC)	0.72	0.38–1.36	0.32
Nonplatinum regimen	0.71	0.41–1.21	0.21
Carboplatin or oxaliplatin	0.75	0.33–1.72	0.50
No chemotherapy	0.32	0.16–0.61	0.001

a CVD, cardiovascular disease; CVD encompasses coronary artery disease, peripheral vascular disease, a history of myocardial infarction, or a cerebrovascular accident; CVD risk factors analyzed include diabetes mellitus, hypertension, and hyperlipidemia.

b Investigator‐designated moderate to severe renal dysfunction.

c Unknown = 22 (3 events).

SHR, subdistribution hazard ratio.

### Survival

**Figure 3:**
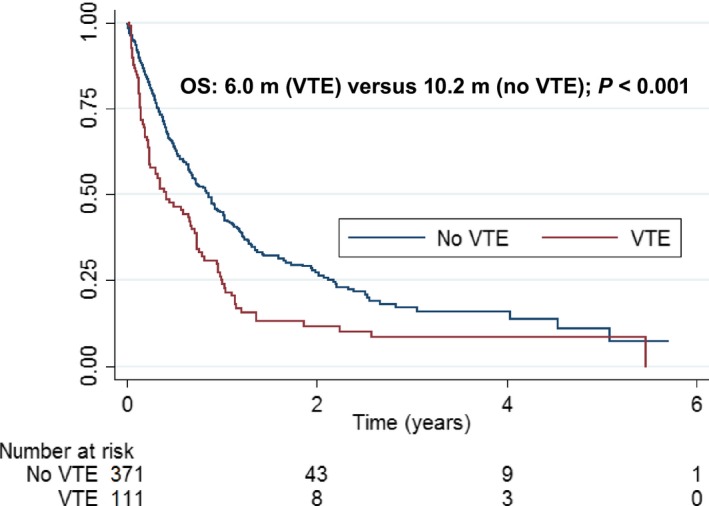
Kaplan–Meier survival curve of patients who had a venous thromboembolic event (VTE) and no venous thromboembolic event (no VTE). *P*‐value is a result of the log‐rank test. OS, overall survival (in months).

There was a statistically significant increase in mortality in patients who had a VTE compared to patients without a VTE (*P* < 0.001). Median survival was 6.0 months (95% CI: 3.1–8.8) from time of VTE versus 10.2 months (95% CI: 8.4–12.2) in matched controls (Fig. 3).

## Discussion

Few studies have examined the VTE incidence rate of patients with metastatic UC/VH. A Dutch population‐based study demonstrated a 6‐month VTE incidence rate of 3.1% in bladder or ureteral cancer patients with distant metastases 17. Recently, Sandhu et al. found a 6.3% 2‐year cumulative VTE incidence rate in patients with advanced bladder cancer utilizing a state cancer registry that was linked to hospital discharge data 18. In our patient cohort, metastatic UC/VH patients had higher cumulative and 6‐month VTE incidence rates, 8.2% and 5.1% respectively, than these previous studies. Importantly, the VTE rate seen in our analysis is comparable to the most thrombogenic primary tumor sites. When compared to the VTE rate (expressed in VTEs per 1000 person‐years) in a systematic review and meta‐analysis of 8 cancer types from 38 studies, the rate in our cohort (75/1000 person‐years) was only superseded by the VTE rate in pancreatic (102/1000 person‐years) and brain cancer (116/1000 person‐years) 6. Consequently, our study supports considering patients with metastatic UC/VH at relatively high risk for VTE.

Venous thromboembolism has been shown to be independently associated with worse overall survival in cancer patients when compared to cancer patients without a VTE. Sørensen et al. reported a 1‐year overall survival of 12% in cancer patients with VTE compared to 36% in matched control cancer patients across a wide range of malignancies 3. Furthermore, other studies, not specifically in UC/VH, have demonstrated that prognosis is worse irrespective of cancer grade or stage 19, 20. Accordingly, the inferior overall survival in metastatic UC/VH patients who had a VTE compared to those who did not in our analysis is consistent with prior studies, and identification of appropriate preventative interventions that may improve outcomes is imperative.

Currently, based on randomized controlled data, routine primary prophylaxis is not recommended in the outpatient oncology setting with the exception of multiple myeloma patients on lenalidomide‐ or thalidomide‐containing regimens 21. One reason for this recommendation is the relatively low frequency of VTEs seen in randomized control trials (RCTs). Two primary prophylaxis RCTs, PROTECHT and SAVE‐ONCO, demonstrated a statistically significant decrease in VTE in multiple solid tumors 13, 22. However, the placebo arms in PROTECHT and SAVE‐ONCO had a VTE rate of only 2.9% and 3.4%, respectively, at a median duration of treatment of approximately 3.5 months. Therefore, the absolute reduction in VTE was too small to justify primary prophylaxis. The absolute incidence in our study (3.4% at 3 months) is similar to the rates seen in PROTECHT and SAVE‐ONCO, thus it is logical to conclude that primary prophylaxis of all patients with metastatic UC/VH would not be recommended.

We evaluated multiple clinical characteristics to identify a subset of metastatic UC/VH patients who might be at particularly high risk for VTE. An association was found between patients with non‐urothelial histology, renal dysfunction, or CVD or CVD risk factors and VTE. However, the underlying mechanism of these associations has not been clearly elucidated. It is likely that non‐urotheilal histology results in more VTEs, at least in part, because patients tend to present with more locally advanced disease when compared to urothelial histology, leading to extrinsic compression of the vasculature and venous thrombosis 23. Several large, epidemiologic studies have shown a link between CVD or CVD risk factors and renal dysfunction with venous clot formation in noncancer patients 24, 25, 26. Patients with CVD or CVD risk factors and/or renal dysfunction may be predisposed to VTE through alterations in the balance of hemostatic proteins. A recent case–control study suggested that chronic kidney disease patients with a VTE had increased levels of Factor VIII and von Willebrand factor 27. Additionally, patients with coronary artery disease and peripheral arterial disease have been shown to have elevated markers of coagulation, such as fibrinogen and D‐dimer, though correlation with VTE rates were not assessed in these studies 28, 29. We believe that the association of VTE with these clinical characteristics has merit, but our findings require validation in an independent cohort. If validated as risk factors, these features may be used in the future to identify a patient population with sufficiently high VTE risk for a primary prophylaxis study.

Numerous studies have suggested that cisplatin increases VTE risk 7, 8, 9. Recently, the combination of GC has been associated with especially high VTE rates. Plimack et al. presented a vascular (arterial and venous) event rate of 23% in a neoadjuvant study of dose‐dense GC for muscle‐invasive bladder cancer, leading to early study closure 30. Thus, we were interested in comparing the VTE rates for GC to other chemotherapy regimens in patients with metastatic UC/VH. In our patient cohort, there was no statistical difference in VTE risk based on chemotherapy group when compared to GC. However, our result may represent a false negative as the analysis was underpowered to detect a small difference. Interestingly, though not statistically significant, the absolute VTE incidence rate was highest in the patients that were treated with GC, with an 11.0% VTE rate. Conversely, patients treated with cisplatin regimens, which did not include gemcitabine, had a VTE rate of 6.9%. Moreover, the point estimates of the subdistribution hazard ratios for each of the chemotherapy groups in the multivariate analysis were <1 when compared to GC. This suggests the possibility of gemcitabine increasing VTE risk or a synergistic effect in patients treated with GC. The decreased incidence of VTE seen in patients who did not receive chemotherapy may support the role of cytotoxic chemotherapy as a risk factor for VTE. However, this result should be interpreted with caution. Patients who did not receive chemotherapy may have had less intensive surveillance imaging, since there was no reason to ascertain treatment response, thereby missing incidental pulmonary emboli.

There are several limitations to our analysis. First, our study is limited by the inherent confounders and biases associated with any retrospective analysis. Second, whether a VTE was catheter associated was not consistently captured and therefore not included in the analysis. Also, data were not available on whether patients were already on anticoagulation for an alternative indication (e.g., atrial fibrillation). Furthermore, some of the clinical factors were investigator designated (e.g., moderate to severe renal dysfunction, diabetes, or hypertension) as opposed to utilizing established criteria for diagnosis of these conditions. We also did not have the percentages of each histologic subtype composing tumors of mixed histologies; therefore, we categorized patients based on the predominant histologic pattern. Finally, during the era in which the data were captured, clinical trials including agents that may increase VTE rates (e.g., bevacizumab, cetuximab) were ongoing and trial participants were not excluded from this analysis 10, 11.

Based on our results, patients with metastatic UC/VH should be counseled about their risk of VTE and the associated clinical signs and symptoms. Patients with significantly increased risk should be considered for primary prophylaxis in clinical trials designed to enroll a high‐risk population. Additionally, larger population‐based studies should be undertaken to validate the clinical risk factors identified in this analysis and further evaluate the impact of chemotherapy regimens on VTE risk. Finally, more intensive investigation of the underlying biologic mechanisms is necessary to improve our understanding of the pathogenesis of cancer‐associated thrombosis and identification of novel biomarkers to improve VTE risk stratification.

## Conflict of Interest

None declared.

## Supporting information


**Table S1.** Chemotherapy regimens utilized in each treatment group.
**Table S2.** Clinical characteristics assessed in univariate analysis for association with VTE risk.
