# Supplementary material for: Venous thromboembolism in metastatic urothelial carcinoma or variant histologies: incidence, associative factors, and effect on survival
Source: Cancer Med. 2016 Dec 20;6(1):186–94. doi: 10.1002/cam4.986 (PMC5269690; doi:10.1002/cam4.986)
Supplement: Supplementary file 1 — Table S1. Chemotherapy regimens utilized in each treatment group. Table S2. Clinical characteristics assessed in univariate analysis for association with VTE risk. [file CAM4-6-186-s001.docx]

SUPPLEMENTARY MATERIAL

**Supplementary Table 1:** Chemotherapy regimens utilized in each treatment group

| **Treatment Group and Regimen Name** | **N (%)** |
| --- | --- |
| **Cisplatin combination (excluding GC)** | **N=202 (%)** |
| Dose-dense MVAC | 82 (40.6) |
| MVAC | 72 (35.6) |
| Cisplatin/etoposide | 14 (6.9) |
| Cisplatin/larotaxel | 11 (5.5) |
| **Non-platinum regimens** | **N=258 (%)** |
| Gemcitabine | 78 (30.2) |
| Paclitaxel | 38 (14.7) |
| Gemcitabine/paclitaxel | 31 (12.0) |
| Docetaxel | 20 (7.8) |
| Sunitinib | 20 (7.8) |
| **Carboplatin or oxaliplatin** | **N=75 (%)** |
| Carboplatin/paclitaxel | 35 (46.7) |
| Carboplatin/etoposide | 17 (22.7) |
| Carboplatin | 9 (12.0) |
| Methotrexate/carboplatin/vinblastine | 8 (10.7) |

*Only chemotherapy regimens used in at least 5% of patients for each

treatment group are included.

# GC=gemcitabine and cisplatin

¥ MVAC=methotrexate, vinblastine, doxorubicin, and cisplatin

**Supplementary Table 2:** Clinical characteristics assessed in univariate analysis for association with VTE risk

| **Clinical Characteristics** |
| --- |
| Age  Race  Gender  ECOG performance status  Prior history of VTE  Body mass index  Moderates to severe renal dysfunction┴  Cardiovascular disease (CVD) or CVD risk factors┼  Albumin  Hemoglobin  White blood cell count  Platelet count  Primary tumor location  Primary tumor histology  Metastases only to non-regional lymph nodes  Presence of liver metastases  Prior perioperative chemotherapy  Surgery* within 2 months of development of metastatic disease  Treatment of the primary tumor with radiotherapy  Number of cycles of first-line chemotherapy |

┴Investigator-designated moderate to severe renal dysfunction

┼CVD = cardiovascular disease; CVD encompasses coronary artery disease, peripheral vascular disease, a history of myocardial infarction, or a cerebrovascular accident; CVD risk factors analyzed include diabetes mellitus, hypertension, and hyperlipidemia.

*****Encompasses patients who underwent a radical cystectomy, nephroureterectomy, nephrectomy, ureterectomy, or urethrectomy..
